# Microglia-Derived Olfactomedin-like 3 Promotes Pro-Tumorigenic Microglial Function and Malignant Features of Glioma Cells

**DOI:** 10.3390/ijms222313052

**Published:** 2021-12-02

**Authors:** Ryan G. Toedebusch, Christopher A. Lucchesi, Eshetu T. Debebe, Luke A. Wittenburg, Xinbin Chen, Christine M. Toedebusch

**Affiliations:** Department of Surgical and Radiological Sciences, School of Veterinary Medicine, University of California, Davis, CA 95616, USA; rgtoed@ucdavis.edu (R.G.T.); calucchesi@ucdavis.edu (C.A.L.); etdebebe@ucdavis.edu (E.T.D.); lwittenburg@ucdavis.edu (L.A.W.); xbchen@ucdavis.edu (X.C.)

**Keywords:** microglia, glioblastoma, olfactomedin-like 3, TGFβ

## Abstract

Under the influence of transforming growth factor-beta (TGFβ), glioma-associated microglia produce molecules that promote glioma growth and invasion. Olfactomedin-like 3 (*Olfml3*), a novel, secreted glycoprotein, is known to promote several non-CNS cancers. While it is a direct TGFβ1 target gene in microglia, the role of microglia-derived OLFML3 in glioma progression is unknown. Here, we tested the hypotheses that microglial *Olfml3* is integral to the pro-tumorigenic glioma-associated microglia phenotype and promotes glioma cell malignancy. Using an *Olfml3* knockout microglial cell line (N9), we demonstrated that *Olfml3* is a direct target gene of all TGFβ isoforms in murine microglia. Moreover, loss of *Olfml3* attenuated TGFβ-induced restraint on microglial immune function and production of cytokines that are critical in promoting glioma cell malignancy. Importantly, microglia-derived OLFML3 directly contributes to glioma cell malignancy through increased migration and invasion. While exposure to conditioned medium (CM) from isogenic control microglia pre-treated with TGFβ increased mouse glioma cell (GL261) migration and invasion, this effect was abolished with exposure to CM from TGFβ-treated *Olfml3*^-/-^ microglia. Taken together, our data suggest that *Olfml3* may serve as a gatekeeper for TGFβ-induced microglial gene expression, thereby promoting the pro-tumorigenic microglia phenotype and glioma cell malignancy.

## 1. Introduction

Glioblastoma (GBM) is the most common and aggressive primary brain tumor of adults, with a 5-year survival rate of approximately 5% [1]. Although immunotherapy has advanced the treatment of non-central nervous system (CNS) tumors, it has failed to overcome the substantial barrier of immune resistance in the glioma microenvironment. While immunoresistance and tumor progression are conferred by a confluence of factors, glioma-associated microglia/macrophages (GAM) play a critical role. As the most abundant infiltrating cells [2], GAM infiltration has been positively correlated with glioma grade [3], invasiveness [4], and resistance to therapy [5,6].

While the glioma-GAM signaling axis is complex, transforming growth factor-beta (TGFβ) isoforms have been recognized to substantially influence the pro-tumorigenic effects of GAM. β1 stimulates GAM to produce cytokines and growth factors promoting glioma growth [7] and invasion [8,9], whereas β2 suppresses GAM immune responses [10]. β3 promotes tumor invasion and augments β1 and β2 signaling [11]. A variety of approaches to inhibit TGFβ signaling [12] and glioma recruitment of GAM [13] have failed to show therapeutic efficacy in GBM [12], underscoring the need for refined therapeutic targets.

Intriguingly, β1 was recently found to induce transcription of a novel gene, *Olfml3*, in mouse microglia. *Olfml3*, encoding the secreted glycoprotein olfactomedin-like 3 (OLFML3), is expressed in microglia but not macrophages [14]. Following exposure to β1, primary mouse microglia increased *Olfml3* mRNA expression 20-fold. Although the role of *Olfml3* in microglia function is unknown, olfactomedin-family proteins modulate the Wnt pathway [15,16], which plays an important role in microglial phenotype determination [17] and gliomagenesis [18]. Importantly, OLFML3 has broad relevance to cancer progression. OLFML3 is a disease biomarker in colon cancer [19] and has been shown to promote neoangiogenesis [20,21], epithelial-to-mesenchymal transition [22], and metastasis [22] in several cancers. 

While the role of OLFML3 in GBM has just begun to be explored, depletion of *OLFML3* in human glioma cells reduced GAM infiltration and extended survival in a glioma xenograft mouse model [23]. However, the function of microglia-derived *Olfml3,* and its contribution to the TGFβ-induced pro-tumorigenic GAM phenotype, is unknown. Therefore, this study aimed to (1) define the function of *Olfml3* in microglia phenotype determination and (2) determine the effect of microglia-derived OLFML3 on the malignant phenotype of murine glioma cells. 

## 2. Results

### 2.1. OLFML3 Is Up-Regulated in GBM and Is a TGFβ Target Gene in Microglia

**Figure 1:**
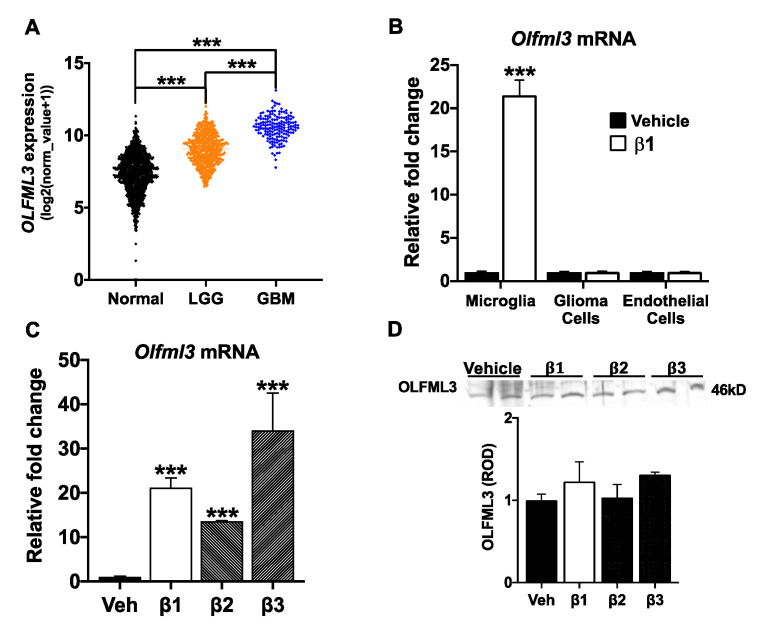
*OLFML3* is increased in GBM and is regulated by TGFβ in microglia. (**A**) *OLFML3* mRNA is increased in low-grade glioma (LGG; *n* = 529) and glioblastoma (GBM; *n* = 166) relative to normal brain (Normal; *n* = 1141) in TCGA patient datasets. (**B**) Exposure to β1 (5 ng/mL; 48 h) increased *Olfml3* mRNA 20-fold in a microglial cell line (N9) but did not affect mRNA expression in a mouse glioma cell line (GL261) or primary mouse brain endothelial cells. Fold was calculated via ΔΔCt and normalized to GAPDH; *** *p* < 0.001. (**C**) Exposure to each TGFβ isoform increased *Olfml3* mRNA (5 ng/mL; 48 h); *** *p* < 0.001. (**D**) Representative immunoblot for OLFML3 protein in N9 cell lysate following exposure to vehicle (Veh) and TGFβ isoforms (5 ng/mL; 48 h). The optical density of OLFML3 protein in cell lysates was measured and normalized to the Ponceau stain. Relative optical densities (ROD) were expressed relative to vehicle-treated cells. No differences were measured between groups (*p* = 0.17). Comparisons based on one-way ANOVA with Tukey’s Multiple Comparison Test. Bars represent group mean with standard error of the mean (SEM); data represent one of three independent experiments.

Examination of The Cancer Genome Atlas (TCGA) transcriptomic datasets revealed that *OLFML3* mRNA expression increased with increasing glioma tumor malignancy. While low-grade gliomas (LGG, *n* = 592) had increased *OLFML3* mRNA levels relative to normal brain ((Normal, *n* = 1141; *p* < 0.001), glioblastomas (GBM, *n* = 166) had increased *OLFML3* mRNA expression relative to both LGG (*p* < 0.001) and normal brain (*p* < 0.001) (Figure 1A). 

To begin to explore putative sources for increased *OLFML3* in GBM, we confirmed *Olfml3* expression in a mouse microglia cell line (N9) [24], a mouse glioma cell line (GL261) [25], and primary mouse brain endothelial cells. As previously demonstrated [26], exposure to β1 increased *Olfml3* mRNA 22-fold in N9 cells relative to vehicle-treated cells (*p* < 0.001). However, neither GL261 nor endothelial cell *Olfml3* mRNA levels were affected by β1 treatment (Figure 1B). Importantly, exposure to all three TGFβ isoforms increased N9 *Olfml3* mRNA (β1: 20-fold, *p* < 0.001; β2: 13-fold, *p* < 0.001; β3: 33-fold, *p* < 0.001) (Figure 1C). Exposure to TGFβ isoforms did not alter OLFML3 protein in N9 cell lysate (*p* = 0.17; Figure 1D). Given these findings, it is possible that increased *OLFML3* mRNA expression in GBM is derived from microglia.

### 2.2. CRISPR/Cas9-Mediated Knockout of Olfml3 in Microglia

**Figure 2:**
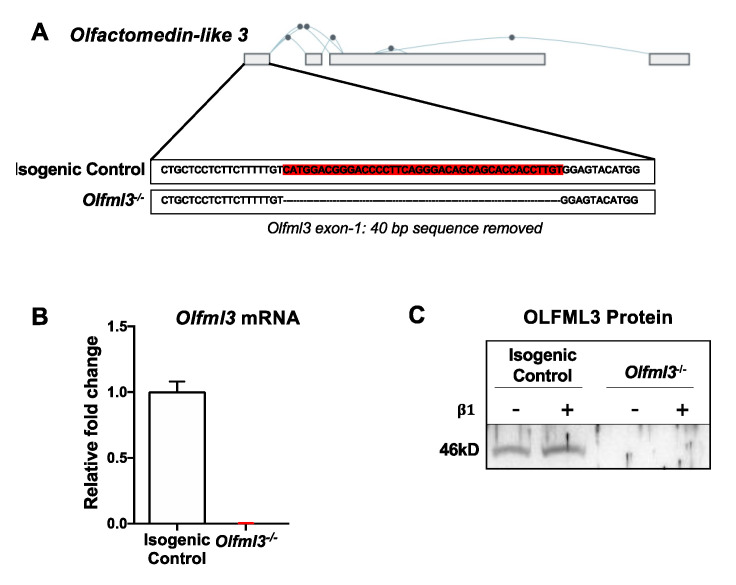
Development and validation of CRISPR-Cas9-mediated *Olfml3* gene editing in microglia. (**A**) Depiction of mouse *Olfml3* with predicted splice variants, demonstrating targeted deletion of 40 bases within Exon 1. (**B**) *Olfml3* mRNA was detected in isogenic control, but not *Olfml3^-/-^,* microglia. (**C**) Representative immunoblot for OLFML3, demonstrating immunoreactivity at the predicted molecular weight (46 kD) in isogenic control, but not *Olfml3^-/-^,* N9 cells. Bars represent group mean with standard error of the mean (SEM); data represent one of three independent experiments.

To determine the function of *Olfml3* and its contribution to the TGFβ-induced pro-tumorigenic phenotype in mouse microglial cells, we performed CRISPR-Cas9-mediated *Olfml3* gene editing in N9 cells. Due to alternative splicing within the *Olfml3* gene (Figure 2A), exon 1 was targeted using the guide RNA, as outlined in Table 1. Forty base-pairs were deleted in Exon 1 (Figure 2A) and verified via Sanger sequencing. This deletion resulted in an immediate stop codon. Knockout of *Olfml3* was validated via qRT-PCR and Western blot (Figure 2B,C). 

### 2.3. Loss of Olfml3 Impaired Microglial Phagocytosis and Chemotaxis 

**Figure 3:**
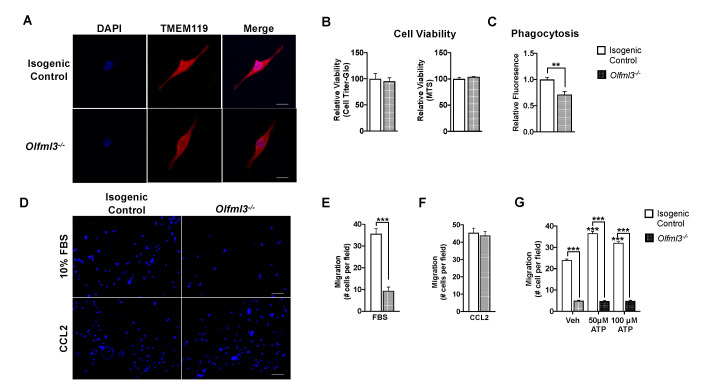
Loss of *Olfml3* impairs microglial phagocytosis and chemotaxis. (**A**) There were no morphological differences detected between isogenic control and *Olfml3^-/-^* microglia; scale bar 5 µm. (**B**) Cellular viability, as assayed by Cell Titer-Glo^®^ (*p* = 0.53) and MTS (*p* = 0.27) assays, was not altered following deletion of *Olfml3* in N9 microglia. (**C**) Microglial phagocytosis of pHrodo^TM^ *Escherichia coli* bioparticles was reduced by 30% in *Olfml3^-/-^* microglia relative to isogenic control cells. Comparisons based on students t-test; ** *p* < 0.01. (**D**) Representative images of isogenic control and *Olfml3^-/-^* microglial migration toward fetal bovine serum (FBS; 10%) or human recombinant C-C motif chemokine ligand 2 (rhCCL2; 10 ng/mL); scale bar 20 µm. (**E**) Loss of *Olfml3* markedly attenuated microglial migration toward FBS relative to isogenic control cells. Comparisons based on students *t*-test; *** *p* < 0.001. (**F**) The rhCCL2 elicited equivalent chemotaxis between isogenic control and *Olfml3^-/-^* N9 cells. Comparisons based on students *t*-test; *p* = 0.54. (**G**) Chemotaxis toward ATP (50 µM, 100 µM) increased relative to vehicle in isogenic control, but not *Olfml3^-/-^*, microglia. Comparisons based on one-way ANOVA with Tukey’s Multiple Comparison Test; *** *p* < 0.001. Bars represent group mean with standard error of the mean (SEM); data represent one of three independent experiments.

Microglia are actively recruited to and proliferate within the glioma microenvironment [27]. Therefore, we evaluated several key microglial functions following *Olfml3* deletion. First, we established that loss of *Olfml3* did not alter microglial morphology (Figure 3A). Moreover, loss of *Olfml3* did not alter cellular viability, as assessed via Cell Titer-Glo^®^ assay (100.0 ± 17.2 vs. 95.4 ± 13.5; *p* = 0.53) and MTS assay (100.0 ± 3.2 vs. 103.9 ± 0.5; *p* = 0.27) (Figure 3B). However, phagocytosis of pHrodo^TM^ *Escherichia coli* bioparticles was reduced in *Olfml3^-/-^* microglia (1.0 ± 0.04 vs. 0.7 ± 0.06; *p* < 0.001) (Figure 3C). Similarly, loss of *Olfml3* altered microglial response to chemotactic cues. Using transwell migration assays, *Olfml3^-/-^* microglia had reduced migration toward fetal bovine serum (FBS; 10%) compared to isogenic control cells (9.4 ± 6 vs. 35.6 ± 7; *p* < 0.001) (Figure 3D,E) but not the potent chemoattractant human recombinant c-c motif chemokine ligand 2 [28] (rhCCL2) (43.7 ± 11 vs. 45.4 ± 10; *p* = 0.54) (Figure 3D,F). Moreover, while isogenic control microglia exhibited increased chemotaxis toward adenosine triphosphate (ATP)-supplemented medium (Veh: 24.4 ± 1 vs. 50 µM: 36.5 ± 1, *p* < 0.0001; 100 µM: 32.3 ± 1, *p* < 0.001) (Figure 3G), this effect was abolished in *Olfml3^-/-^* microglia (50 µM: 4.8 ± 0.2, *p* < 0.001; 100 µM: 4.9 ± 0.3, *p* < 0.001) (Figure 3G). Taken together, these findings suggest that loss of *Olfml3* induces specific perturbations in microglial response to environmental stimuli.

### 2.4. Microglial Olfml3 Is Necessary for the Pro-Tumorigenic GAM Phenotype 

**Figure 4:**
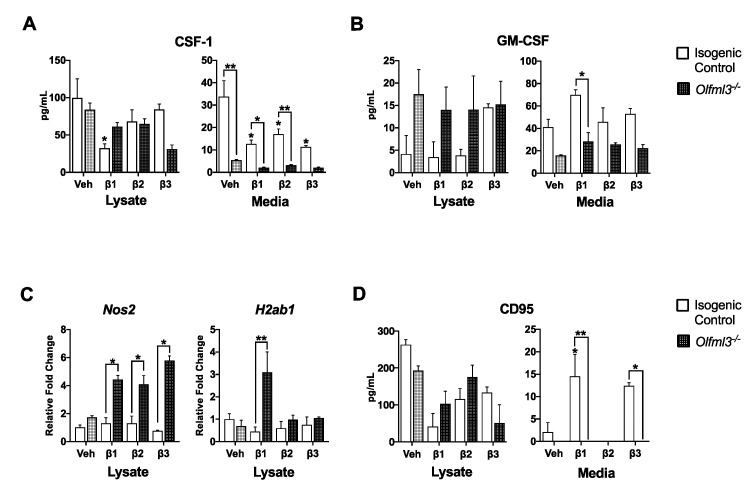
*Olfml3* deletion attenuated TGFβ-induced microglial immunosuppression. (**A**) Secretion of colony stimulating factor-1 (CSF-1) was attenuated in *Olfml3^-/-^* microglia relative to isogenic control microglia in all conditions; * *p* < 0.05, ** *p* < 0.01. (**B**) While exposure to β1 increased secretion of granulocyte–macrophage colony stimulating factor (GM-CSF) in isogenic control microglia, secretion was reduced in *Olfml3^-/-^* microglia; * *p* < 0.05. (**C**) The mRNA levels of the pro-inflammatory genes *Nos2* and *H2ab1* were increased in *Olfml3* microglia relative to isogenic control cells following treatment with TGFβ; * *p* < 0.05, ** *p* < 0.01. (**D**) Secretion of CD95, a potent inducer of cytotoxic T cell apoptosis, increased in isogenic control microglia following exposure to β1 and β3, but was undetectable in the media of *Olfml3^-/-^* microglia across all conditions; * *p* < 0.05, ** *p* < 0.01. Comparisons based on one-way ANOVA with Tukey’s Multiple Comparison Test. Bars represent group mean with standard error of the mean (SEM); data represent one of three independent experiments.

Once recruited to the glioma microenvironment, GAMs provide a major source of cytokines to support glioma growth [29]. In the absence of *Olfml3*, microglial secretion of key cytokines promoting microglial invasion and GBM growth were reduced. Secreted levels of colony stimulating factor-1 (CSF-1), a cytokine critical for microglial recruitment and glioma growth [30], were reduced in *Olfml3^-/-^* microglia media compared to isogenic control media following exposure to vehicle (5.5 ± 0.3 vs. 33.8 ± 7, *p* < 0.01) and TGFβ treatment (β1: 12.6 ± 10 vs. 2.1 ± 0.3, *p* < 0.05; β2: 17.0 ± 4 vs. 3.2 ± 0.4, *p* < 0.01; β3: 11.4 ± 1 vs. 2.1 ± 0.4, *p* = 0.06) (Figure 4A). Similar to CSF-1, granulocyte–macrophage colony stimulating factor (GM-CSF) is a key molecule promoting microglial proliferation [31] and glioma progression [32]. Under the influence of β1, loss of *Olfml3* attenuated secretion of GM-CSF relative to β1-stimulated isogenic control cells (2.2 ± 0.3 vs. 11.9 ± 3; *p* < 0.05) (Figure 4B).

Coinciding with production of tumor supportive cytokines, GAM’s anti-tumor immunity is suppressed in GBM. Remarkably, *Nos2* mRNA, which encodes inducible nitric oxide synthase to generate cytotoxic nitric oxide [33], was increased in *Olfml3^-/-^* microglia relative to isogenic control microglia following exposure to β1 (4-fold; *p* < 0.05), β2 (4-fold; *p* < 0.05), and β3 (6-fold; *p* < 0.01) (Figure 4C). Moreover, *H2-Ab1* mRNA, encoding major histocompatibility class II [34], increased 3-fold in *Olfml3^-/-^* microglia relative to isogenic control microglia following exposure to β2 (*p* < 0.05) (Figure 4C). Microglial secretion of CD95, a Fas ligand, has been implicated in immune evasion and induction of cytotoxic T cell apoptosis [35]. While CD95 was increased in the media of isogenic control cells following exposure to β1 (14.5 ± 5 vs. 2.1 ± 2; *p* < 0.05) and β3 (12.4 ± 1; *p* < 0.05), it remained undetectable in *Olfml3^-/-^* microglial media under all conditions (Figure 4D). 

### 2.5. OLFML3 Promotes Glioma Cell Migration and Invasion

**Figure 5:**
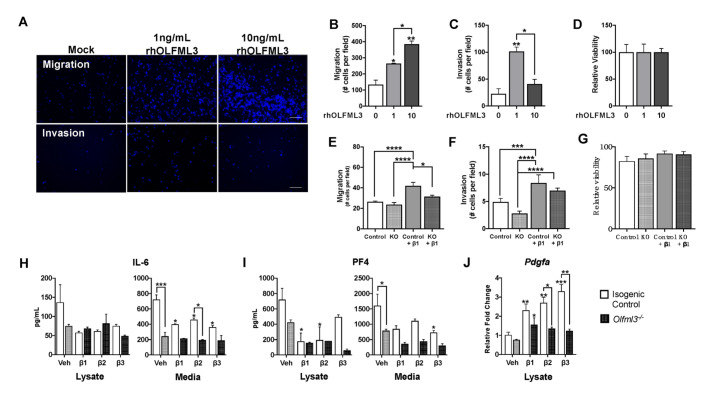
OLFML3 promotes glioma cell migration and invasion. (**A**) Representative images of glioma cell (GL261) migration and invasion following treatment with human recombinant OLFML3 (rhOLFML3; 1 ng/mL, 10 ng/mL); scale bar 20 µm. (**B**) Exposure to rhOLFML3 induced a concentration-dependent increase in GL261 migration relative to vehicle-treated (0) cells (1: 1 ng/mL, 10: 10 ng/mL; 48 h); * *p* < 0.05, ** *p* < 0.01. (**C**) GL261 invasion was increased following exposure to 1 ng/mL, but not 10 ng/mL, rhOLFML3 relative to vehicle-treated cells (48 h); * *p* < 0.05, ** *p* < 0.01. (**D**) Cell viability was not affected by rhOLFML3 (1 ng/mL, 10 ng/mL; 48 h). (**E**) Migration was similar between GL261 cells exposed to conditioned media (CM) from vehicle-treated isogenic control and *Olfml3^-/-^* microglia (48 h). However, exposure to CM from isogenic control, but not *Olfml3^-/-^*, microglia pre-treated with β1 increased GL261 migration (48 h; 5 ng/mL); * *p* < 0.05, **** *p* < 0.0001. (**F**) GL261 invasion was increased following exposure to CM from isogenic control microglia pre-treated with β1 (48 h; 5 ng/mL) relative to CM from vehicle-treated isogenic control and *Olfml3^-/-^* microglia (48 h). Exposure to CM from *Olfml3^-/-^* microglia pre-treated with β1 increased GL261 invasion relative to CM from vehicle-treated *Olfml3^-/-^* microglia; *** *p* < 0.001, **** *p* < 0.0001. (**G**) GL261 viability was not affected by exposure to microglia CM under any condition (*p* = 0.4925). (**H**) Interleukin-6 (IL-6) was reduced in the media of *Olfml3^-/-^* microglia relative to isogenic control microglia following exposure to vehicle and β1; * *p* < 0.05, *** *p* < 0.001. (**I**) Secretion of platelet factor 4 (PF4) was attenuated in *Olfml3^-/-^* microglia following exposure to vehicle and TGFβ isoforms; * *p* < 0.05. (**J**) Loss of microglial *Olfml3* abolished the TGFβ-induced increase in *Pdgfa* mRNA (48 h; 5 ng/mL); * *p* < 0.05, ** *p* < 0.01, *** *p* < 0.001. Comparisons based on one-way ANOVA with Tukey’s Multiple Comparison Test. Bars represent group mean with standard error of the mean (SEM); data represent one of three independent experiments.

To determine the effect of OLFML3 on the glioma cell malignancy, we exposed GL261 mouse glioma cells to recombinant human OLFML3 (rhOLFML3) for 48 h. Using transwell assays, we observed a dose-dependent increase in GL261 migration following exposure to rhOLFML3 (Figure 5A,B). Exposure to 1 ng/mL rhOLFML3 increased GL261 migration compared to vehicle-treated cells (134.1 ± 19 vs. 265.1 ± 0.6; *p* < 0.05) (Figure 5B). Migration was further increased following exposure to 10 ng/mL rhOLFML3 (384.3 ± 14; *p* < 0.001) (Figure 5B). Similarly, GL261 invasion was increased following exposure to 1 ng/mL rhOLFML3 compared to vehicle-treated cells (22.6 ± 7 vs. 101.4 ± 6; *p* < 0.001) (Figure 5A,C). However, this effect was lost following exposure to 10 ng/mL rhOLFML3 (40.6 ± 6 vs. 22.6 ± 7; *p* = 0.25) (Figure 5C). Interestingly, rhOLFML3 did not act as a chemoattract for GL261 cells, as neither GL261 migration nor invasion was altered by rhOLFML3-supplemented medium in the bottom chamber of a transwell assay (Supplemental Figure S1). Moreover, GL261 viability was not affected by exposure to rhOLFML3 (100.0 ± 6 vs. 100.0 ± 7 vs. 100.0 ± 3; *p* = 0.14) (Figure 5D).

To determine the contribution of TGFβ-induced, microglia-derived OLFML3 on GL261 migration and invasion, GL261 cells were exposed to conditioned medium (CM) from isogenic control and *Olfml3^-/-^* microglia following vehicle or β1 pre-treatment (5 ng/mL; 48 h). Migration was similar between GL261 cells exposed to CM from vehicle-treated isogenic control and *Olfml3^-/-^* microglia (26.4 ± 0.8 vs. 23.6 ± 2; *p* = 0.84) (Figure 5E). As expected, GL261 migration increased following exposure to CM from isogenic control microglia pre-treated with β1 vs. vehicle (42.0 ± 3; *p* < 0.0001) (Figure 5E). However, loss of *Olfml3* abolished this effect, as migration rates were similar between GL261 cells exposed to CM from β1 pre-treated *Olfml3^-/-^* microglia (31.6 ± 1) and vehicle-treated isogenic control (*p* = 0.42) and *Olfml3^-/-^* microglia (*p* = 0.09) (Figure 5E). Similarly, GL261 invasion was increased following exposure to CM from isogenic control microglia pre-treated with β1 vs. vehicle (4.9 ± 0.7 vs. 8.4 ± 2; *p* < 0.001) (Figure 5F). Again, this effect was abolished in the absence of microglial *Olfml3*, with similar invasion rates between GL261s treated with CM from β1-treated *Olfml3^-/-^* microglia and vehicle-treated isogenic control microglia (6.9 ± 0.5 vs. 4.9 ± 0.7; *p* = 0.06) (Figure 5F). Cellular viability was not affected by exposure to microglial CM under any condition (81.3 ± 1 vs. 91.0 ± 5; *p* > 0.999) (Figure 5G).

In addition to the loss of OLFML3 in microglial CM on GL261 malignancy, *Olfml3* deletion significantly reduced microglial secretion of key cytokines that promote GBM invasion. While there were no differences in cell lysate concentrations, secretion of interleukin-6 (IL-6) was markedly attenuated in *Olfml3^-/-^* microglia compared to isogenic control cells following exposure to vehicle (244.6 ± 49 vs. 721.5 ± 61; *p* < 0.001) and β2 (193.8 ± 7 vs. 458.4 ± 21; *p* < 0.05) (Figure 5H). Moreover, secretion of platelet factor 4 (PF4), a growth factor critical in GBM invasion [36], was reduced following vehicle treatment in *Olfml3^-/-^* microglia compared to isogenic control cells (426.0 ± 437 vs. 721.2 ± 206; *p* < 0.05) (Figure 5I). Similarly, loss of microglial *Olfml3* abolished the TGFβ-induced increase in *Pdgfa* mRNA, a key negative prognostic indicator in GBM [37] (β1: 2.3 ± 0.6 vs. 1.6 ± 0.6, *p* < 0.05); β2: 2.7 ± 0.5 vs. 1.4 ± 0.1; β3: 3.3 ± 0.6 vs. 1.2 ± 0.2, *p* < 0.01) (Figure 5J). 

## 3. Discussion

In this study, we began to uncover the role of *Olfml3* in microglial function and glioma cell malignancy. Our data showed that microglial *Olfml3* is a direct target gene of all TGFβ isoforms and plays a key role in TGFβ-induced, pro-tumorigenic microglia phenotype determination. Importantly, our data suggest that OLFML3 may directly contribute to glioma cell malignancy through increasing migration and invasion capacity. The myriad pro-tumorigenic effects of microglia-derived *Olfml3* illuminates the potential for therapeutic development targeting the TGFβ-GAM-*Olfml3* signaling axis in GBM. 

OLFML3 is a secreted glycoprotein that belongs to the family of the olfactomedin domain-containing proteins [15]. It has been identified as an extracellular matrix protein [20], suggesting that the majority of OLFML3 is secreted. This aligns well with our observation that TGFβ exposure dramatically increases *Olfml3* mRNA but not protein expression in the cell lysate. 

While the biological function of olfactomedin domain-containing proteins remains incompletely characterized, growing evidence indicates that they are important for intercellular signaling and protein–protein interaction during development and disease. In particular, olfactomedin 4 (OLFM4), a member of a closely related subfamily of OLFML3, negatively regulates pro-inflammatory responses. OLFM4 knockout mice have enhanced bacterial clearance of *Staphylococcus aureus* and *Escherichia coli* through modulation of neutrophil killing [38], as well as *Helicobacter pylori* through disinhibition of NF-kB [39]. Moreover, *Olfm4* deletion exacerbated inflammation and mucosal damage in a mouse model of colitis [40], further supporting its role in immune restraint. Similarly, our study suggests that *Olfml3* may restrict microglial immune responses, thereby contributing to the markedly immunosuppressed tumor microenvironment of GBM.

Anti-tumor immune responses in GBM are limited through the combination of GAM and T cell dysfunction. Within the glioblastoma microenvironment, GAMs exert immunosuppressive functions through direct cell–cell interactions and release of soluble factors. Importantly, microglia function as antigen-presenting cells in the CNS, requiring up-regulation of MHC II for T cell activation [41]. However, this activity is suppressed in GBM [34]. In fact, MHC I and MHC II molecules were absent in 50% of GBM samples [42], with specific suppression of GAM MHC II occurring through TGFβ signaling. In line with these findings, we demonstrated that *Olfml3* deletion abolished β1-mediated transcriptional suppression of MHC II, which may improve microglial antigen presentation function. Additionally, loss of *Olfml3* may mitigate T cell turnover. In the glioma microenvironment, GAM perpetuate CD4^+^/CD8^+^ T cell apoptosis through secretion of CD95 [35], the ligand for the T cell death receptor Fas, and IL-6, a potent inducer of Fas [43]. Strikingly, *Olfml3* deletion abolished microglial secretion of CD95. While exposure to TGFβ increased secretion in isogenic control cells, CD95 was undetectable in the media of *Olfml3^-/-^* in all conditions. Moreover, loss of *Olfml3* attenuated secretion of IL-6. These findings, coupled with the dependency of microglial *Olfml3* expression upon TGFβ1-SMAD2-mediated de novo protein synthesis [26], suggest that *Olfml3* functions as a gatekeeper for TGFβ-induced effects on microglia-mediated immunity.

Importantly, targeting the immunomodulatory effects of *Olfml3* may enhance efficacy of currently available immunotherapies. Expression of the immune checkpoint molecule programmed cell death ligand-1 (PD-L1) is inversely correlated with overall patient survival in GBM [44]. While there are many ongoing Phase I and II clinical trials targeting PD-1/PD-L1, preliminary results in patients with recurrent GBM demonstrate unpredictable efficacy, with meager to no survival benefit compared to standard therapies [45,46,47]. As IL-6 is necessary and sufficient for PD-L1 induction [48], we speculate that therapeutic targeting of *Olfml3* may enhance current immunotherapeutic approaches for GBM patients. In support of this hypothesis, recent work has demonstrated that anti-OLFML3 therapy in conjunction with anti-PD1 immunotherapy increased overall survival in a mouse model of colorectal cancer [21]. Thus, inhibition of microglial *Olfml3*, in tandem with immune checkpoint blockade, may yield improved patient survival in GBM. 

Treatment resistance is also governed by the diffuse infiltrative capacity of glioblastoma. Our results support the hypothesis that microglia-derived OLFML3 acts as a paracrine factor facilitating glioma cell invasion. Glioma cell migration and invasion were only affected following 48 h exposure to rhOLFML3, suggesting that OLFML3 may regulate key signaling pathways in glioma cells. This is consistent with general properties of the olfactomedin protein family, which are known to interact with multiple protein binding partners and regulate several cell signaling pathways [16]. This effect is in contrast to recent work that demonstrated that glioma-derived OLFML3 is a GAM chemoattractant [23]. Thus, OLFML3 may have cell type-specific functions within the glioma microenvironment that collectively support tumor growth. Moreover, OLFML3 expression is likely regulated by multiple molecules. The circadian regulator CLOCK and its partner BMAL1 have been identified to promote transcriptional upregulation of OFLML3 in GBM cells [23]. Remarkably, TGFβ signaling is necessary for normal circadian clock function [49]. In fact, TGFβ induces expression of the core clock gene *Per1* [50]. The interaction between *CLOCK*, *BMAL1,* and molecules of the canonical TGFβ signaling pathway in GBM is unknown. However, it is interesting to consider the interconnectedness of these systems and their possible synergistic promotion of OLFML3 expression in microglia and glioma cells alike.

Herein, our data demonstrated that microglia-derived *Olfml3* may contribute to glioma cell malignancy through intrinsic and extrinsic mechanisms. Silencing of *Olfml3* attenuated the pro-tumorigenic microglial secretome, as well as mitigating glioma cell malignancy in vitro. Together, these results provide a rationale for further exploration of anti-OLFML3 therapeutic strategies in GBM. 

## 4. Materials and Methods

### 4.1. Cell Culture and Reagents

The N9 microglial cell line [24] was generously donated from Jyoti Watters at The University of Wisconsin School of Veterinary Medicine. N9 cells were submitted to ATCC for authentication and confirmed to be of murine origin. N9 cells were maintained in DMEM (Gibco^TM^, ThermoFisher Scientific, Waltham, MA, USA) supplemented with 10% fetal bovine serum (FBS) (Gibco^TM^, ThermoFisher Scientific, Waltham, MA, USA) and 1% Penicillin/Streptomycin (Gibco^TM^, ThermoFisher Scientific, Waltham, MA, USA). The GL261 mouse glioma cell line was obtained from the Developmental Therapeutics Program Repository at the National Cancer Institute. GL261 cells were maintained in RPMI 1640 (Gibco^TM^, ThermoFisher Scientific, Waltham, MA, USA) supplemented with 10% fetal bovine serum (FBS) (Gibco^TM^, ThermoFisher Scientific, Waltham, MA, USA). All cells were confirmed to be Mycoplasma-free and maintained at 37 °C in a humidified incubator with 5% CO_2_. All cells were used below passage 15 and within 1 month after thawing. 

### 4.2. CRISPR/Cas9-Mediated Olfml3 Knockout 

Generation of the *Olfml3*-knockout (*Olfml3^-/-^*) microglial cell line was achieved using the CRISPR-Cas9 gene editing system. All reagents were purchased from Integrated DNA Technologies (IDT; Coralville, IA, USA) and used according to the manufacturer’s recommendations. Briefly, a guide-RNA (Table 1), targeted to exon 1 of *Olfml3* and the tracrRNA-ATTO-550, was duplexed and mixed with recombinant Cas9 enzyme (IDT, Coralville IA, USA) to form the ribonucleoprotein (RNP) complex. The RNP complex was transfected into cells using Lipofectamine CRISPRMAX (ThermoFisher Scientific, Waltham, MA, USA) transfection reagent. Then, 24 h following transfection, cells were subjected to fluorescence-activated cell sorting and individual ATTO-550-positive cells were sorted into a single well of a 96-well plate. Each single cell created a clonal population, whereby Sanger sequencing confirmed *Olfml3* editing within the defined region of exon 1. Western blot analysis confirmed successful *Olfml3* knockout. An isogenic control line was generated using the same parameters described above without the addition of the gRNA for *Olfml3*. 

### 4.3. Human Recombinant OLFML3 Protein Generation

The protein sequence for OFLML3, consisting of 406 amino acids, is 94.3% identical between human and mouse as determined by a protein BLAST through the National Center for Biotechnology Information. The OLFML3 sequence was cloned into pTXB1 Vector (NEB, N6707S) using *Olfml3* cDNA (Addgene, Wattertown, MA, USA) as template with the following primers: forward, 5’-GGTGGTCATATGGGGCCCAGCACCCCT-3’, and reverse, 5’-GGTGGTTGCTCTTCCGCAAACCTCCTCCTCTTTCTTCCTCAT-3’. The pTXB1-OLFML3 vector was electroporated into ClearColi^®^ BL21 (DE3) Electrocompetent cells (Lucigen, Middleton, WI, USA). These cells have a genetically modified Lipopolysaccharide (LPS) that does not trigger endotoxic response in subsequent assays. Briefly, pTXB1-OLFML3-expressing ClearColi cells were induced (500 μM Isopropyl β-d-1-thiogalactopyranoside (IPTG)) at OD600 = 0.72 and incubated at 16 °C for 18 h. Cells were pelleted, lysed, and incubated with Chitin resin. After washing the beads, rhOLFML3 was cleaved using 50 mM Dithiothreitol (DTT) at 4 °C for 72 h. The rhOLFML3 was eluted and concentrated using Pierce™ Protein Concentrator PES column, 10,000 Da molecular weight cutoff (ThermoFisher Scientific, Waltham, MA, USA). The rhOLFML3 protein was subjected to Fast Protein Liquid Chromatography (FPLC) using HIPREP 16/60 SEPHACRYL S-200 column to remove residual DTT before BCA quantification and subsequent use in all experiments. 

### 4.4. Generation of Anti-OLFML3 Antibody

We generated an anti-OLFML3 polyclonal antibody using the commercially available service from Cocalico Biologicals (Reamstown, PA, USA). Briefly, recombinant OLFML3 protein was generated as described above, purified, and electrophoresed on a 12% SDS-PAGE gel. The OLFML3 band was excised and sent to Cocalico for inoculation of rabbit host. Serum antibody titer was tested until endogenous OLFML3 was detectable using wild-type N9 microglia and rhOLFML3 as a positive control. Final exsanguination was carried out and antibody was purified from the final serum volume. 

### 4.5. Quantitative Real-Time PCR

Cells were grown to 80% confluency and treated with human recombinant TGFβ isoforms (5 ng/mL; β1: 100–21, PeproTech, Cranbury, NJ, USA; β2: PHG9114, Life Technologies, ThermoFisher Scientific, Waltham, MA, USA; β3: SRP3171, Sigma-Aldrich, St. Louis, MO, USA) or vehicle (PBS; ThermoFisher Scientific, Waltham, MA, USA) once every 24 h for a total of two treatments (48-h total incubation). We evaluated *Olfml3* mRNA expression in murine microglia cells following 24-, 48-, and 72-h exposure to TGFβ. We observed the greatest increase in *Olfml3* mRNA at 48 h; thus, all subsequent experiments were performed at this timepoint. Cells were pelleted and RNA was isolated with the Direct-zol MiniPrep kit (Zymo Research, Irvine, CA, USA) according to manufacturer’s specifications. Using one microgram-purified DNase-treated RNA, cDNA was reverse transcribed using the High-Capacity cDNA Reverse Transcription Kit (Thermo Fisher—Applied Biosystems, ThermoFisher Scientific, Waltham, MA, USA). Primer sets were designed using NCBI primer design (https://www.ncbi.nlm.nih.gov/tools/primer-blast/index.cgi) (accessed on 4 August 2019) and purchased through IDT (Table 1). Primer validation was performed using a 4× cDNA serial dilution series from isogenic control microglia as template. The efficiency and fit of the generated curves were evaluated; primer sets that did not produce efficiency of at least 0.9 and R^2^ value of 0.95 from the cDNA dilution series were rejected. Only experimental quantification cycle (Cq) values that fell within the boundaries of the validated curves were used for analysis.

The qPCR reactions consisted of primer pairs at a final concentration of 200 nM, 50 ng cDNA template, and 2× SSoAdvanced Universal SYBR Green Superix (Bio-Rad, Hercules, CA, USA) per manufacturer’s protocol on a CFXConnect (Bio-Rad, Hercules, CA, USA) machine as previously described [51]. All reactions were run as 20-µL triplicates, and the average Cq was used as the data point for a given sample. The mRNA expression values were quantified by the 2^−ΔΔCt^ method, whereby ΔCT = 18S Ct−gene of interest Ct.

### 4.6. Immunofluorescence and Confocal Microscopy

Isogenic control and *Olfml3^-/-^* microglia were cultured on sterile glass coverslips treated with fibronectin. Cells were fixed using 4% (*w/v*) paraformaldehyde (Millipore Sigma, Burlington, MA, USA), washed three times for 5 min at RT, and permeabilized with 0.1% Triton X-100-Tris-buffered saline (TBST) for 15 min at RT and blocked for 2 hours at RT with normal goat serum (5% *w/v*) and bovine serum albumin (1% *w/v*) in TBST. Cells were incubated in primary antibody solution (mouse monoclonal anti-TMEM119 (BioLegend, San Diego, CA, USA #853302; 1:1000) in fresh blocking buffer) overnight at 4 °C. Cells were washed three times for 5 min at RT and incubated in secondary antibody solution for 1 hour at RT (IgG (heavy and light) anti-mouse Alexa Fluor 555 (Molecular Probes, Invitrogen, Carlsbad, CA, USA; 1:1000) in fresh blocking buffer). Cells were washed three times for 5 min at RT and mounted with Vectashield with 4′5-diamidino-2phenylindole (DAPI) (Vector Labs, Burlingame, CA, USA). Images were captured via Leica TCS Sp8 STED 3× confocal microscope.

### 4.7. Western Blot Analysis

Whole cell protein samples were lysed using RIPA buffer (50 mM Tris–HCl (pH 8.0), 150 mM NaCl, 1% NP-40, 0.5% sodium deoxycholate, and 1% SDS, 1× protease/phosphatase inhibitor cocktail (Thermo Fisher Scientific, Waltham, MA, USA)). The cellular homogenate was rotated for 30 min at 4 °C and centrifuged at 12,000× *g* for 10 min. Protein concentrations of the resultant supernatants were determined using the BCA assay (Pierce Biotechnology, Rockford, IL, USA). Forty micrograms of protein were loaded, electrophoresed on 15% SDS-PAGE gels, and transferred to nitrocellulose membranes overnight. All blots were incubated with Ponceau S (Sigma, St. Louis, MO, USA) to validate equal loading and transfer across all lanes. Membranes were blocked overnight at 4 °C in 5% fat-free milk. Anti-OLFML3 primary antibody was diluted (1:1000) in Tris-buffered saline + Tween-20 (TBST) with 1% fat-free milk and applied to the membrane overnight at 4 °C with gentle rocking. The membranes were washed three times in TBST and incubated in horseradish peroxidase (HRP)-conjugated secondary antibody (1:20,000; Cell Signaling Technology, Danvers, MA, USA) for 1 h at RT with gentle rocking. The HRP substrate for enhanced chemiluminescence (ThermoFisher Scientific, Waltham, MA, USA) was applied immediately prior to exposure. Band densitometry was performed using Image Lab (Bio-Rad, Hercules, CA, USA) and normalized to the Ponceau as a protein loading and transfer control. Optical densities were normalized to vehicle-treated conditions and expressed as relative optical densities (ROD). All experiments were independently repeated in triplicate.

### 4.8. Murine Protein Arrays

Isogenic control and *Olfml3^-/-^* microglia were grown to 80% confluency and treated with human recombinant TGFβ isoforms as described above (5 ng/mL; 48 h), followed by serum starvation for 12 h (0.1% FBS). The cell media were aspirated, centrifuged at 350 g for 5 min, and concentrated using Pierce PES protein concentrator columns (ThermoFisher Scientific, Waltham, MA, USA). Whole cell protein samples were treated as described for Western blot. Cell lysate and media samples were sent for analysis by RayBiotech Life (Peachtree Corners, GA, USA) with standard quality control. In brief, Quantibody® Mouse Full Testing Service (QAH-INF-1) utilized two non-overlapping arrays of antibody pairs to quantify selected molecules. RayBiotech confirmed no cross reactivity between antibody pairs and standard controls.

### 4.9. Cell Viability

Cell viability was performed using the Cell Titer Glo® 2.0 Assay (Promega, Madison, WI, USA) according to the manufacturer’s protocol. Cells (2.5 × 10^4^) were seeded in 96-well, black-sided plates. Titer Glo® reagent was added to each well and the plate was incubated for 10 min at RT on a plate shaker, followed by luminescence recording via plate reader (BioTek800TS). Optical densities were recorded for six replicates per condition and the average optical density of media alone (blank) was subtracted from all experimental conditions. Three independent experiments were performed. 

Similar to Cell Titer Glo®, cells (2.5 × 10^4^) were seeded in 96-well, black-sided plates and cultured for 48 h. MTS reagent was added to each well and the plate was incubated for 10 min at RT on a plate shaker, followed by absorbance reading via plate reader (BioTek800TS) at 590 mm. Optical densities were recorded for six replicates per condition. Three independent experiments were performed. 

### 4.10. Transwell Migration and Invasion Assays

The modified Boyden chamber assay was used for analysis of cell migration and invasion. Migration assays were performed using cells (microglia: 2 × 10^5^; GL261: 5 × 10^4^) suspended in serum-free culture medium and seeded into 24-well Transwell inserts with an 8-µm pore polycarbonate filter insert. Invasion assays were conducted similarly, with the addition of 50-µL Matrigel coating onto the 8-µm pore polycarbonate filter insert. FBS, serum-free medium with indicated factors (rhCCL2: 479-JE-010, R&D Systems; ATP: A6419-1G, Sigma, St. Louis, MO, USA) or CM, was added to the receiver wells. After 90 min (ATP) or 24 h (rhCCL2, FBS), inserts were removed and the top of each insert was swabbed to remove non-migrated cells. Remaining cells attached to the bottom of the insert were fixed using 4% paraformaldehyde. Membranes were excised from the inserts and mounted onto microscope slides using mounting medium containing 4′,6- diamidino-2-phenylindole (DAPI). Nine photographs were taken per membrane, with three technical replicates per experiment, using a brightfield microscope (Leica, DM5000 B). Cells were identified by positive DAPI immunoreactivity and quantified via an ImageJ custom macro. Three independent experiments were performed. 

### 4.11. Phagocytosis Assay

Isogenic control and *Olfml3^-/-^* microglia were seeded in 96-well plates (2 × 10^5^) and incubated overnight. The following day, media were removed and cells were incubated with pHrodo™Green E.Coli BioParticles (ThermoFisher Scientific, Waltham, MA, USA) for 1 h at 37 °C following the manufacturer’s protocol. Luminescence was determined via microplate reader (Molecular Devices SpectraMax Gemini EM Microplate Reader 19745) at 509/533 nm. Percent phagocytosis was calculated as follows:

(1) $$\%\;\mathrm{phagocytosis}\;=\frac{\mathrm{net}\;\mathrm{experimental}\;\mathrm{phagocytosis}\;\times 100\%}{\mathrm{net}\;\mathrm{positive}\;\mathrm{control}\;\mathrm{phagocytosis}}$$

### 4.12. Statistics

Statistical analysis was performed with Prism GraphPad V9.0.2 software. Data are presented as the mean ± SEM. Cell culture experiments were performed in technical replicates, with three biological replicates. Data were tested for normality via Shapiro–Wilks test. Statistical significance was assessed via unpaired two-tailed Student’s *t*-test or ANOVA with Tukey’s multiple comparisons test. Results were regarded as statistically significant for *p* < 0.05.

## Figures and Tables

**Table 1:** Primer and guide-RNA sequences used for PCR and CRISPR-Cas9, respectively.

Gene.	Sequence-F (5′ to 3′)	Sequence-R (5′ to 3′)	Use
O*lfml3* s1	GCTAACGGGCTGGAGGGAAA	AGTGGTACCATCCCATCCGA	PCR
O*lfml3* s2	AGCTGCCTTAGAGGAACGG	CCTCCCTTTCAAGACGGTCC	qPCR
*H2-Ab1*	AGCCCCATCACTGTGGAGT	GATGCCGCTCAACATCTTGC	qPCR
*Nos2*	TTCTCAGCCACCTTGGTGAAG	AAGTGAAATCCGATGTGGCC	qPCR
*Pdgfa*	GAGGAAGCCGAGATACCCC	TGCTGTGGATCTGACTTCGAG	qPCR
*Rpl22*	AGCAGGTTTTGAAGTTCACCC	CAGCTTTCCCATTCACCTTGA	qPCR
*Olfml3-gRNA*	TCATGGACGGGACCCCTTCA		CRISPR

## Data Availability

Not applicable.

## Supplementary Materials

The following are available online at https://www.mdpi.com/article/10.3390/ijms222313052/s1.
