# Supplementary material for: Microglia-Derived Olfactomedin-like 3 Promotes Pro-Tumorigenic Microglial Function and Malignant Features of Glioma Cells
Source: Int J Mol Sci. 2021 Dec 2;22(23):13052. doi: 10.3390/ijms222313052 (PMC8657851; doi:10.3390/ijms222313052)
Supplement: Supplementary file 1 [file ijms-22-13052-s001.zip › ijms-1465229-supplementary.pdf]

International Journal of
Molecular Sciences

Supplementary Materials

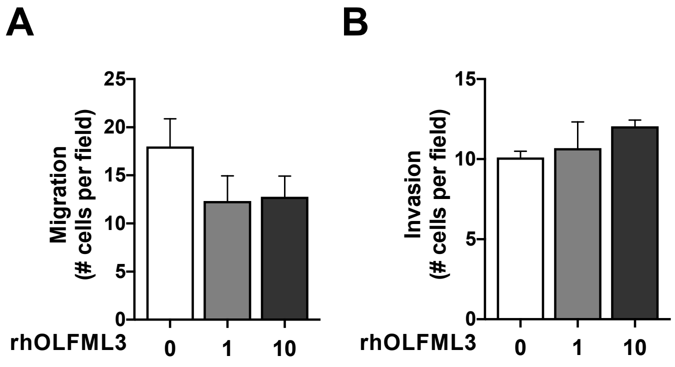

Figure S1. OLFML3 is not a chemoattractant for glioma cells. rhOFLML3-supplemented medium (1: 1 ng/mL, 10: 10 ng/mL) in the bottom chamber of a transwell assay did not affect GL261 (**A**) migration (p = 0.29), nor (**B**) invasion (p = 0.53). Comparisons based on one-way ANOVA with Tukey's Multiple Comparison Test. Bars represent group mean with standard error of the mean (SEM).
